# Genome-wide identification of soybean WRKY transcription factors in response to salt stress

**DOI:** 10.1186/s40064-016-2647-x

**Published:** 2016-06-29

**Authors:** Yanchong Yu, Nan Wang, Ruibo Hu, Fengning Xiang

**Affiliations:** The Key Laboratory of Plant Cell Engineering and Germplasm Innovation, School of Life Sciences, Shandong University, Jinan, 250100 Shandong China; Shandong Key Laboratory of Plant Biotechnology, College of Life Sciences, Qingdao Agricultural University, Qingdao, 266109 Shandong China; Qingdao Institute of Bioenergy and Bioprocess Technology, Chinese Academy of Sciences, Songling Road No. 189, Qingdao, 266101 Shandong China

**Keywords:** WRKY, Soybean, Expression patterns, Salt stress

## Abstract

Members of the large family of WRKY transcription factors are involved in a wide range of developmental and physiological processes, most particularly in the plant response to biotic and abiotic stress. Here, an analysis of the soybean genome sequence allowed the identification of the full complement of 188 soybean WRKY genes. Phylogenetic analysis revealed that soybean WRKY genes were classified into three major groups (I, II, III), with the second group further categorized into five subgroups (IIa–IIe). The soybean WRKYs from each group shared similar gene structures and motif compositions. The location of the *GmWRKYs* was dispersed over all 20 soybean chromosomes. The whole genome duplication appeared to have contributed significantly to the expansion of the family. Expression analysis by RNA-seq indicated that in soybean root, 66 of the genes responded rapidly and transiently to the imposition of salt stress, all but one being up-regulated. While in aerial part, 49 *GmWRKYs* responded, all but two being down-regulated. RT-qPCR analysis showed that in the whole soybean plant, 66 *GmWRKYs* exhibited distinct expression patterns in response to salt stress, of which 12 showed no significant change, 35 were decreased, while 19 were induced. The data present here provide critical clues for further functional studies of WRKY gene in soybean salt tolerance.

**Electronic supplementary material:**

The online version of this article (doi:10.1186/s40064-016-2647-x) contains supplementary material, which is available to authorized users.

## Background

Soybean (*Glycine max*) is a global cash crop. Apart from its major contribution to human and animal nutrition, the seed provides a feedstock for biodiesel production and represents a significant raw material for a number of pharmaceutical and industrial processes (Phang et al. 2008; Wang et al. 2010). In recent years, the demand for soybean is increasing rapidly, so it attracted more and more attention to improve soybean agronomic traits, such as stress tolerance. Soybean productivity is greatly compromised by soil salt. However, during the long period of evolution, soybean has evolved complex strategies to survive salt stress. These strategies are originated from the changes of various aspects, such as the genome, gene expression, metabolism and physiology (Phang et al. 2008). To present, functionally reported salt tolerance related genes in soybean are mainly categorized into several classes, including ion transporter coding genes (e.g. *GmHKT1*, *GmSALT3*, *GmNHX1*, *GmCAX1* and *GmCHX1*) (Chen et al. 2011; Guan et al. 2014; Li et al. 2006; Luo et al. 2005a; Qi et al. 2014), transcription factors (TFs) (e.g. GmNAC11/-20/-29, GmDREB1, GmMYB76/-92/-174/-177, GmbZIP44/-62/-78, GmWRKY27 and GmERF7) (Hao et al. 2011; Jin et al. 2010; Liao et al. 2008a, b; Wang et al. 2015; Zhai et al. 2013) and others genes (e.g. glutathione S-transferase gene *GsGST*, late embryogenesis abundant gene *GmLEA*, calcineurin B-like protein coding gene *GmCBL1* and flavone synthase gene *GmFNSII*) (Ji et al. 2010; Lan et al. 2005; Li et al. 2012; Phang et al. 2008; Wang et al. 2011; Yan et al. 2014; Zhou et al. 2010).

The product of a TF gene binds to a specific *cis*-regulatory sequence(s) in the promoter of its target gene. The WRKYs are among the largest class of plant TFs, and their promoter target (the W-box) has the sequence (T)(T)TGAC(C/T) (Rushton et al. 2010). WRKY TFs are recognized by the presence of a conserved DNA-binding region composed of about 60 residues (the “WRKY” domain), which harbors the WRKYGQK heptapeptide followed by a C_2_H_2_ or C_2_HC zinc finger motif (Rushton et al. 2010). In some cases, the heptapeptide can take the form WRKYGKK or WRKYGEK (Rushton et al. 2010). WRKY TFs have been classified into three main groups: those possessing two heptapeptides are clustered into group I; both group I and II members harbor one C_2_H_2_ type zinc finger motif, while the group III members feature a C_2_HC one. The large size of group II has been addressed by its division into five subgroups (IIa, IIb, IIc, IId and IIe), based on peptide sequence (Eulgem et al. 2000; Rushton et al. 2010).

Since the first reports of WRKY TFs (dePater et al. 1996; Ishiguro and Nakamura 1994; Rushton et al. 1995, 1996), considerable progress has been made in revealing the function of WRKY TFs. They are elucidated to be involved in various developmental and physiological processes (Rushton et al. 2010), such as seed development (Lagace and Matton 2004; Luo et al. 2005b), seed dormancy and germination (Xie et al. 2005, 2007; Zentella et al. 2007), senescence (Miao et al. 2004; Robatzek and Somssich 2002; Ulker et al. 2007) trichome morphogenesis (Johnson et al. 2002), metabolic pathways (Kato et al. 2007; Sun et al. 2003) and plant development (Cai et al. 2014; Devaiah et al. 2007; Guo et al. 2015; Yu et al. 2013, 2016). The particularly prominent roles of WRKY in plant appear to be the modulation of response to biotic and abiotic stresses (Chen et al. 2012; Eulgem and Somssich 2007; Pandey and Somssich 2009). In the root of the model plant *Arabidopsis*, 18 WRKY genes have been shown to be induced by exposure to salt stress (Jiang and Deyholos 2006); In rice, ten WRKY genes (of 13 analyzed) respond differentially to a range of abiotic stress treatment (Qiu et al. 2004), while in *Brachypodium distachyon*, over 60 % of a set of 86 WRKY genes assayed were up-regulated by heat and cold stress and over 50 % were down-regulated by salt, drought and/or oxidation stress (Wen et al. 2014). Among the soybean WRKY TFs, 25 out of 64 have been shown to be differentially expressed in response to at least one abiotic stress treatment (Zhou et al. 2008).

Base on the availability of the complete soybean genome sequence and several databases (PlantTFDB, SoyDB, SoyTFKB, NCBI and Phytozome), a previous study reported a genome-wide characterization of the WRKY family in soybean and a functional analysis of some genes involved in response to *Phakopsora pachyrhizi* (Bencke-Malato et al. 2014). However, in Phytozome (release v10), the new assembly (v2.0) replaces the Glyma1 assembly. The new database corrects several issues in pseudomolecule reconstruction in the Glyma1 assembly. According to the new database and a recent RNA-Seq result (Belamkar et al. 2014), Song et al. identified 176 GmWRKYs and analyzed their expression files in different tissues and in response to drought and salt stress (Song et al. 2016). However, beside Phytozome, there are many other soybean genome sequence databases. Furthermore, several other transcriptome experiment data sets of soybean under abiotic stress are provided by NCBI website. Here, integrating more databases and RNA-Seq results (Belamkar et al. 2014; Wei et al. 2015), we made a new genome-wide identification of soybean WRKYs and compared their response to salt stress in different tissues. In addition, we analyzed expression profiles of 66 *GmWRKYs* by quantitative RT-PCR (RT-qPCR). Our findings provide new clues for further investigation of WRKY gene in soybean salt tolerance.

## Methods

### Retrieval of GmWRKY sequences

A set of 185 *GmWRKY* sequences was recovered from Phytozome (http://phytozome.jgi.doe.gov/pz/portal.html, release 10.2) using the keyword PF03106 as a search term, along with three further *GmWRKY* sequences from the NCBI database (http://www.ncbi.nlm.nih.gov). The presence of a WRKY domain(s) in all 188 *GmWRKYs* was confirmed by running the SMART program (http://smart.embl-heidelberg.de) (Letunic et al. 2015). These *GmWRKY* genes were further checked in PlantTFDB (http://planttfdb.cbi.pku.edu.cn/, release 3.0) (Jin et al. 2014) and SoyTFKB (http://www.igece.org/Soybean_TF/).

### Multiple sequence alignment and phylogenetic analysis

A multiple alignment of the *WRKY* sequences was performed using the ClustalW program implemented in MEGA v6.06 software package (http://www.megasoftware.net/) (Tamura et al. 2013). The sequences were also subjected to a phylogenetic analysis using the neighbor-joining method; the resulting tree was based on 1000 bootstrap replicates, the p-distance model and pairwise deletion.

### Gene structure and conserved motifs analysis

The exon–intron structure of each gene was derived by comparing its coding sequence with the corresponding genomic DNA sequence, using the GSDS program (http://gsds.cbi.pku.edu.cn/) (Hu et al. 2015). The online program MEME v4.10.1 (http://meme-suite.org/tools/meme) was used to identify the conserved motifs present; the relevant parameters were: number of repetitions = any; maximum number of motifs = 16; optimum width of each motif = 6–70 residues.

### Genomic location and gene duplication

Each WRKY gene was positioned in the genome by reference to the full genome sequence. The gene duplications in *GmWRKY* genes were identified based on the investigations described in previous study (Schmutz et al. 2010), and Circos software was used to provide a graphical representation of the position of homeologous chromosome segments (Krzywinski et al. 2009).

### RNA-seq analysis

A transcriptomic analysis was based on archival RNA-seq data collected from a set of salt stress experiments, mounted on the NCBI GEO database (Belamkar et al. 2014). Experiments GSM1377923, -24 and -25 represented three independent replicates of plants sampled before any exposure to salt; GSM1377935, -36 and -37 related to plants sampled after a 1 h exposure to the stress; GSM1377938, -39 and -40 after a 6 h exposure; and GSM1377941, -42 and -43 after a 12 h exposure. The other transcriptomic analysis was also based on archival RNA-seq data derived from the NCBI GEO database (Wei et al. 2015), experiment GSE57960 was related to plants sampled after a 12 h exposure to salt stress, the aerial part of plants was used for sequencing. The reads per kilobase of exon model per million mapped reads (RPKM) algorithm was used for normalization and mean normalized values were used for the analysis. The transcription response was given in the form of fold changes relative to the 0 h control. Cluster v3.0 software (University of Tokyo, Human Genome Center) was used to perform hierarchical clustering, which was visualized using Java TreeView software (Saldanha 2004). The relevant parameters were: similarity measurement: correlation (uncentered); linkage method: average linkage method.

### Plant materials

Seed of *cv*. Williams 82 was germinated on a sheet of moist filter paper, and the seedlings were grown under a regime of 28/20 °C, 14 h photoperiod, light intensity 800 μmol m^−2^ s^−1^ and relative humidity 55 %. Two weeks old seedlings were exposed to 200 mM NaCl for either 0, 2, 6 or 24 h, after which the whole seedling was harvested, snap-frozen in liquid nitrogen and stored at −80 °C.

### RT-qPCR analysis

Total RNA was extracted from the frozen plant material using the TRIzol reagent (Invitrogen, USA) following to the manufacturer’s instructions. The resulting RNA was treated with RNase-free DNaseI (Promega, USA) to remove genomic DNA contamination, and the cDNA First strand was synthesized with 3 μg total RNA by TransScript One-step gDNA Removal and cDNA synthesis SuperMix (TransGen, China) following the manufacturer’s protocol. The subsequent RT-qPCRs and data analysis were performed using a Bio-Rad Real-Time PCR detection system (Bio-Rad) based on the SYBR Green I master mix, as reported previously (Bustin et al. 2009; Seo et al. 2009). According to a previous study, *GmELF1b* was most stably expressed under salt stress, so it was used as a reference gene (Le et al. 2012). All reactions were carried out in triplicate, using samples harvested from independent plants. The relevant primer sequences are given in Additional file 1: Table S1.

## Results and discussion

### Identification of WRKY genes in soybean

The WRKYs represent one of the largest families of plant TFs. The acquisition of full genome sequences has simplified the enumeration of *WRKY* copy number, so that it is now clear that there are 81 *WRKY* copies in tomato (Huang et al. 2012), 55 in cucumber (Ling et al. 2011), 104 in poplar (He et al. 2012), 59 in grapevine (Wang et al. 2014), 116 in cotton (Dou et al. 2014) and 119 in maize (Wei et al. 2012). In a previous study, 182 putative WRKY gene models were identified (Bencke-Malato et al. 2014). In recent, phytozome updated the soybean assembly; the new assembly (v2.0) replaced the Glyma1 assembly. Therefore, we performed a comprehensive analysis of soybean WRKY sequences obtained from Phytozome, PlantTFDB and NCBI, and finally identified a total of 185 non-redundant putative WRKY genes. Compared with previous 182 WRKY genes, six genes *GmWRKY49* (*Glyma.05G203900*), *GmWRKY53* (*Glyma.06G061900*), *GmWRKY72* (*Glyma.07G161100*), *GmWRKY108* (*Glyma.10G171000*), *GmWRKY130* (*Glyma.14G085500*) and *GmWRKY131* (*Glyma.14G100100*) are novel ones, while three previous genes *GmWRKY17* (*Glyma06g06530*), *GmWRKY38* (*Glyma18g48460*) and *GmWRKY132* (*Glyma14g11440*) are considered obsolete according to the current version of the annotated genome. However, the three obsolete genes have been retained for further study. Thus, a total of 188 annotations of GmWRKYs were presented in this study (Table 1). All the 188 retrieved sequences were proved to contain WRKY domains using SMART analysis.

**Table 1:** The WRKY gene family in Soybean

Gene name	Gene ID^a^	Conserved heptapeptide^b^	Group	Chromosome	Amino acid
*GmWRKY1*	Glyma.01G043300	WRKYGQK	IIb	Chr1	509
*GmWRKY2*	Glyma.01G053800	WRKYGQK/WRKYGQK	I	Chr1	455
*GmWRKY3*	Glyma.01G056800	WRKYGQK	IIc	Chr1	297
*GmWRKY4*	Glyma.01G128100	WRKYGEK/WRKYGQK	I	Chr1	507
*GmWRKY5*	Glyma.01G189100	WRKYGQK	IId	Chr1	321
*GmWRKY6*	Glyma.01G222300	WRKYGQK	IIe	Chr1	245
*GmWRKY7*	Glyma.01G224800	WRKYGQK	III	Chr1	322
*GmWRKY8*	Glyma.02G007500	WRKYGQK	IIb	Chr2	484
*GmWRKY9*	Glyma.02G010900	WRKYGQK	IIc	Chr2	320
*GmWRKY10*	Glyma.02G020300	WRKYGQK	IIb	Chr2	480
*GmWRKY11*	Glyma.02G112100	WRKYGQK/WRKYGQK	I	Chr2	455
*GmWRKY12*	Glyma.02G115200	WRKYGQK	IIc	Chr2	293
*GmWRKY13*	Glyma.02G141000	WRKYGQK	IId	Chr2	355
*GmWRKY14*	Glyma.02G203800	WRKYGQK/WRKYGQK	I	Chr2	505
*GmWRKY15*	Glyma.02G232600	WRKYGQK/WRKYGQK	I	Chr2	580
*GmWRKY16*	Glyma.02G285900	WRKYGQK	IIc	Chr2	337
*GmWRKY17*	Glyma.02G293400	WRKYGQK	IIb	Chr2	401
*GmWRKY18*	Glyma.02G297400	WRKYGQK/WRKYGQK	I	Chr2	588
*GmWRKY19*	Glyma.02G306300	WRKYGQK/WRKYGQK	I	Chr2	507
*GmWRKY20*	Glyma.03G002300	Lost	III	Chr3	271
*GmWRKY21*	Glyma.03G042700	WRKYGEK/WRKYGQK	I	Chr3	507
*GmWRKY22*	Glyma.03G109100	WRKYGQK	IIc	Chr3	238
*GmWRKY23*	Glyma.03G159700	WRKYGQK	IId	Chr3	341
*GmWRKY24*	Glyma.03G176600	WRKYGQK/WRKYGQK	I	Chr3	448
*GmWRKY25*	Glyma.03G220100	WRKYGQK	IId	Chr3	253
*GmWRKY26*	Glyma.03G220800	WRKYGQK	IIc	Chr3	287
*GmWRKY27*	Glyma.03G224700	WRKYGQK	IIb	Chr3	541
*GmWRKY28*	Glyma.03G256700	WRKYGQK	III	Chr3	362
*GmWRKY29*	Glyma.04G054200	WRKYGKK	IIc	Chr4	161
*GmWRKY30*	Glyma.04G061300	WKKYGQK	IIa	Chr4	222
*GmWRKY31*	Glyma.04G061400	WRKYGQK	IIa	Chr4	220
*GmWRKY32*	Glyma.04G076200	WRKYGQK	IId	Chr4	279
*GmWRKY33*	Glyma.04G115500	WRKYGQK/WRKYGQK	I	Chr4	761
*GmWRKY34*	Glyma.04G173500	WRKYGQK	IIb	Chr4	531
*GmWRKY35*	Glyma.04G218400	WRKYGQK	IIc	Chr4	234
*GmWRKY36*	Glyma.04G218700	WRKYGKK	IIc	Chr4	196
*GmWRKY37*	Glyma.04G223200	WRKYGQK	III	Chr4	337
*GmWRKY38*	Glyma.04G223300	WRKYGQK	III	Chr4	317
*GmWRKY39*	Glyma.04G238300	WRKYGQK	III	Chr4	364
*GmWRKY40*	Glyma.05G029000	WRKYGQK	IIb	Chr5	594
*GmWRKY41*	Glyma.05G096500	WRKYGQK	IId	Chr5	334
*GmWRKY42*	Glyma.05G123000	WRKYGQK	IIb	Chr5	361
*GmWRKY43*	Glyma.05G123600	WRKYGQK	IIe	Chr5	430
*GmWRKY44*	Glyma.05G127600	WRKYGQK	IIc	Chr5	358
*GmWRKY45*	Glyma.05G160800	WRKYGQK	IIe	Chr5	255
*GmWRKY46*	Glyma.05G165800	WRKYGKR	III	Chr5	1355
*GmWRKY47*	Glyma.05G184500	WRKYGKK	IIc	Chr5	188
*GmWRKY48*	Glyma.05G185400	WRKYGQK	IIc	Chr5	216
*GmWRKY49*	Glyma.05G203900	Lost	IIc	Chr5	99
*GmWRKY50*	Glyma.05G211900	WRKYGQK	IIe	Chr5	288
*GmWRKY51*	Glyma.05G215900	WRKYGQK	III	Chr5	363
*GmWRKY52*	Glyma.06G054500	WRKYGKK	IIc	Chr6	175
*GmWRKY53*	Glyma.06G061900	WRKYGQK	IIa	Chr6	309
*GmWRKY54*	Glyma06g06530*	WRKYGQK	IIa	Chr6	294
*GmWRKY55*	Glyma.06G077400	WRKYGQK	IId	Chr6	300
*GmWRKY56*	Glyma.06G125600	WRKYGQK	III	Chr6	364
*GmWRKY57*	Glyma.06G142000	WRKYGQK	III	Chr6	319
*GmWRKY58*	Glyma.06G142100	WRKYGQK	III	Chr6	331
*GmWRKY59*	Glyma.06G147100	WRKYGKK	IIc	Chr6	196
*GmWRKY60*	Glyma.06G147500	WRKYGQK	IIc	Chr6	236
*GmWRKY61*	Glyma.06G168400	WRKYGKK	IIc	Chr6	160
*GmWRKY62*	Glyma.06G190800	WRKYGQK	IIb	Chr6	615
*GmWRKY63*	Glyma.06G212900	WKKYGQK	IIa	Chr6	242
*GmWRKY64*	Glyma.06G219800	WRKYGQK/WRKYGQK	I	Chr6	470
*GmWRKY65*	Glyma.06G242200	Lost/WRKYGQK	I	Chr6	176
*GmWRKY66*	Glyma.06G307700	WRKYGQK	IIb	Chr6	628
*GmWRKY67*	Glyma.06G320700	WRKYGQK/WRKYGQK	I	Chr6	776
*GmWRKY68*	Glyma.07G023300	WRKYGQK	IIa	Chr7	311
*GmWRKY69*	Glyma.07G057400	WRKYGQK	III	Chr7	369
*GmWRKY70*	Glyma.07G116300	WRKYGQK	IIc	Chr7	237
*GmWRKY71*	Glyma.07G133700	WRKYGQK	IId	Chr7	317
*GmWRKY72*	Glyma.07G161100	Lost/WRKYGQK	I	Chr7	252
*GmWRKY73*	Glyma.07G227200	WRKYGQK/WRKYGQK	I	Chr7	533
*GmWRKY74*	Glyma.07G238000	WRKYGQK	IIc	Chr7	391
*GmWRKY75*	Glyma.07G262700	WRKYGQK	IIb	Chr7	576
*GmWRKY76*	Glyma.08G011300	WRKYGEK	IIc	Chr8	147
*GmWRKY77*	Glyma.08G018300	WRKYGQK	IIe	Chr8	292
*GmWRKY78*	Glyma.08G021900	WRKYGQK	III	Chr8	359
*GmWRKY79*	Glyma.08G078100	WRKYGQK	IIb	Chr8	181
*GmWRKY80*	Glyma.08G078700	WRKYGQK	IIe	Chr8	429
*GmWRKY81*	Glyma.08G082400	WRKYGQK	IIc	Chr8	371
*GmWRKY82*	Glyma.08G118200	WRKYGQK	IIe	Chr8	261
*GmWRKY83*	Glyma.08G142400	WRKYGKK	IIc	Chr8	184
*GmWRKY84*	Glyma.08G143400	WRKYGQK	IIc	Chr8	235
*GmWRKY85*	Glyma.08G218600	WRKYGQK	IIa	Chr8	313
*GmWRKY86*	Glyma.08G240800	WRKYGQK/WRKYGQK	I	Chr8	523
*GmWRKY87*	Glyma.08G320200	WRKYGQK	IIb	Chr8	486
*GmWRKY88*	Glyma.08G325800	WRKYGQK/WRKYGQK	I	Chr8	577
*GmWRKY89*	Glyma.09G005700	WRKYGQK	IIb	Chr9	541
*GmWRKY90*	Glyma.09G029800	WRKYGQK	IIe	Chr9	506
*GmWRKY91*	Glyma.09G034300	WRKYGQK	IIc	Chr9	331
*GmWRKY92*	Glyma.09G061900	WRKYGQK	IId	Chr9	296
*GmWRKY93*	Glyma.09G080000	WRKYGQK	IIb	Chr9	458
*GmWRKY94*	Glyma.09G127100	WRKYGQK	IIb	Chr9	242
*GmWRKY95*	Glyma.09G129100	WRKYGQK	IIe	Chr9	372
*GmWRKY96*	Glyma.09G240000	WRKYGQK	IIb	Chr9	541
*GmWRKY97*	Glyma.09G244000	WRKYGQK	IIc	Chr9	238
*GmWRKY98*	Glyma.09G250500	WRKYGQK/WRKYGQK	I	Chr9	734
*GmWRKY99*	Glyma.09G254400	WRKYGQK	IIc	Chr9	192
*GmWRKY100*	Glyma.09G254800	WRKYGQK	IIe	Chr9	348
*GmWRKY101*	Glyma.09G274000	WRKYGQK	III	Chr9	300
*GmWRKY102*	Glyma.09G280200	WIKYGQK/WRKYGQK	I	Chr9	543
*GmWRKY103*	Glyma.10G011300	WRKYGQK	IIc	Chr10	323
*GmWRKY104*	Glyma.10G032900	WRKYGQK	IId	Chr10	392
*GmWRKY105*	Glyma.10G111400	Lost	IIb	Chr10	305
*GmWRKY106*	Glyma.10G113800	WRKYGKK	IIa	Chr10	120
*GmWRKY107*	Glyma.10G138300	WRKYGQK	IIb	Chr10	482
*GmWRKY108*	Glyma.10G171000	WHQYGLK	IIc	Chr10	367
*GmWRKY109*	Glyma.10G171100	WRKYGQK	IIc	Chr10	192
*GmWRKY110*	Glyma.10G171200	WRKYGQK	IIc	Chr10	336
*GmWRKY111*	Glyma.10G230200	WRKYGQK	IIe	Chr10	297
*GmWRKY112*	Glyma.11G021200	WRKYGQK	IIe	Chr11	214
*GmWRKY113*	Glyma.11G053100	WRKYGQK	IId	Chr11	321
*GmWRKY114*	Glyma.11G163300	WRKYGQK/WRKYGQK	I	Chr11	548
*GmWRKY115*	Glyma.12G097100	WRKYGQK	IIb	Chr12	614
*GmWRKY116*	Glyma.12G152600	WRKYGQK/WRKYGQK	I	Chr12	467
*GmWRKY117*	Glyma.12G212300	WRKYGQK	IIe	Chr12	263
*GmWRKY118*	Glyma.13G102000	WRKYGQK	IId	Chr13	324
*GmWRKY119*	Glyma.13G117600	WRKYGQK	IIb	Chr13	383
*GmWRKY120*	Glyma.13G267400	WRKYGQK	III	Chr13	294
*GmWRKY121*	Glyma.13G267500	WRKYGQK	III	Chr13	296
*GmWRKY122*	Glyma.13G267600	WRKYGQK	III	Chr13	300
*GmWRKY123*	Glyma.13G267700	WRKYGQK	III	Chr13	270
*GmWRKY124*	Glyma.13G289400	WRKYGQK	IIe	Chr13	265
*GmWRKY125*	Glyma.13G310100	WRKYGQK	IIb	Chr13	614
*GmWRKY126*	Glyma.13G370100	WRKYGQK	IIa	Chr13	309
*GmWRKY127*	Glyma.14G006800	WRKYGQK/WRKYGQK	I	Chr14	508
*GmWRKY128*	Glyma.14G016200	WRKYGQK/WRKYGQK	I	Chr14	585
*GmWRKY129*	Glyma.14G028900	WRKYGQK	IIc	Chr14	335
*GmWRKY130*	Glyma.14G085500	Lost	IId	Chr14	276
*GmWRKY131*	Glyma.14G100100	WRKYGKK	IIc	Chr14	68
*GmWRKY132*	Glyma14g11440*	WRKYGKK	IIc	Chr14	137
*GmWRKY133*	Glyma.14G102900	WRKYGQK	IIa	Chr14	278
*GmWRKY134*	Glyma.14G103100	WRKYGQK	IIa	Chr14	282
*GmWRKY135*	Glyma.14G135400	WRKYGQK	IId	Chr14	316
*GmWRKY136*	Glyma.14G185800	WRKYGQK	III	Chr14	329
*GmWRKY137*	Glyma.14G186000	WRKYGQK	III	Chr14	303
*GmWRKY138*	Glyma.14G186100	WRKYGQK	III	Chr14	240
*GmWRKY139*	Glyma.14G199800	WRKYEDK	III	Chr14	332
*GmWRKY140*	Glyma.14G200200	WRKYGQK/WRKYGQK	I	Chr14	575
*GmWRKY141*	Glyma.15G003300	WRKYGQK	IIa	Chr15	330
*GmWRKY142*	Glyma.15G110300	WRKYGQK	IIb	Chr15	599
*GmWRKY143*	Glyma.15G135600	WRKYGQK	IIe	Chr15	523
*GmWRKY144*	Glyma.15G139000	WRKYGQK	IIc	Chr15	356
*GmWRKY145*	Glyma.15G168200	WRKYGQK	IId	Chr15	293
*GmWRKY146*	Glyma.15G186300	WRKYGQK	IIb	Chr15	451
*GmWRKY147*	Glyma.16G026400	WRKYGQK	III	Chr16	373
*GmWRKY148*	Glyma.16G031400	WRKYGQK	IIc	Chr16	195
*GmWRKY149*	Glyma.16G031900	WRKYGQK	IIe	Chr16	335
*GmWRKY150*	Glyma.16G054400	WRKYGQK	IIc	Chr16	195
*GmWRKY151*	Glyma.16G176700	WRKYGQK	IIe	Chr16	274
*GmWRKY152*	Glyma.16G177000	WRKYGQK	IIe	Chr16	408
*GmWRKY153*	Glyma.16G219800	WRKYGQK	III	Chr16	265
*GmWRKY154*	Glyma.17G011400	WRKYGQK	IIb	Chr17	489
*GmWRKY155*	Glyma.17G035400	WRKYGQK	IIc	Chr17	398
*GmWRKY156*	Glyma.17G042300	WRKYGQK	IIb	Chr17	391
*GmWRKY157*	Glyma.17G057100	WRKYGQK	IId	Chr17	320
*GmWRKY158*	Glyma.17G074000	WRKYGQK/WRKYGQK	I	Chr17	505
*GmWRKY159*	Glyma.17G097900	WRKYGQK	IIb	Chr17	600
*GmWRKY160*	Glyma.17G168900	WRKYGQK	IId	Chr17	332
*GmWRKY161*	Glyma.17G197500	WRKYGQK	IId	Chr17	316
*GmWRKY162*	Glyma.17G222300	WRKYGQK	IIa	Chr17	312
*GmWRKY163*	Glyma.17G222500	WRKYGQK	IIa	Chr17	278
*GmWRKY164*	Glyma.17G224800	WRKYGKK	IIc	Chr17	164
*GmWRKY165*	Glyma.17G239200	WRKYGQK	IId	Chr17	278
*GmWRKY166*	Glyma.18G056600	WRKYGQK/WRKYGQK	I	Chr18	542
*GmWRKY167*	Glyma.18G081200	WRKYGQK/WRKYGQK	I	Chr18	577
*GmWRKY168*	Glyma.18G092200	WRKYGQK	IIb	Chr18	478
*GmWRKY169*	Glyma.18G124700	WRKYGQK	IIb	Chr18	529
*GmWRKY170*	Glyma.18G183100	WRKYGQK	IId	Chr18	308
*GmWRKY171*	Glyma.18G208800	WRKYGQK/WRKYGQK	I	Chr18	541
*GmWRKY172*	Glyma.18G213200	WRKYGQK	III	Chr18	299
*GmWRKY173*	Glyma.18G238200	WRKYGQK	IIe	Chr18	351
*GmWRKY174*	Glyma.18G238600	WRKYGQK	IIc	Chr18	192
*GmWRKY175*	Glyma.18G242000	WRKYGQK/WRKYGQK	I	Chr18	744
*GmWRKY176*	Glyma.18G256500	WRKYGQK	IIb	Chr18	541
*GmWRKY177*	Glyma.18G263400	WRKYGQK/WRKYGQK	I	Chr18	520
*GmWRKY178*	Glyma18g48460*	WRKYGQK	IIc	Chr18	225
*GmWRKY179*	Glyma.19G020600	WRKYGQK	IIb	Chr19	495
*GmWRKY180*	Glyma.19G094100	WRKYGQK	IIc	Chr19	188
*GmWRKY181*	Glyma.19G177400	WRKYGQK/WRKYGQK	I	Chr19	471
*GmWRKY182*	Glyma.19G217000	WRKYGQK	IId	Chr19	264
*GmWRKY183*	Glyma.19G217800	WRKYGQK	IIc	Chr19	290
*GmWRKY184*	Glyma.19G221700	WRKYGQK	IIb	Chr19	516
*GmWRKY185*	Glyma.19G254800	WRKYGQK	III	Chr19	362
*GmWRKY186*	Glyma.20G028000	WRKYGQK/WRKYGQK	I	Chr20	439
*GmWRKY187*	Glyma.20G030500	WRKYGQK	IIb	Chr20	163
*GmWRKY188*	Glyma.20G163200	WRKYGQK	IIe	Chr20	321

^a^Genes that are not annotated in the new assembly (v2.0) are marked with the star symbol

^b^The variants of conserved WRKYGQK peptide are shown in red color and some conserved WRKYGQK sequences are lost in several members

In previous genome-wide studies, the commonly accepted nomenclature for WRKY members was based on their location order on chromosomes (Dou et al. 2014; Ling et al. 2011). Identically, in the present study, *GmWRKYs* was designated from *GmWRKY1* to *GmWRKY188* based on their exact physical position from the top to the bottom on the soybean chromosomes 1–20 (Table 1). For genes producing more than one transcript, only the primary sequence was named. This nomenclature system was different from previous study (Bencke-Malato et al. 2014). A full comparison of currently known WRKY genes is given in Additional file 2: Table S2.

**Fig. 1:**
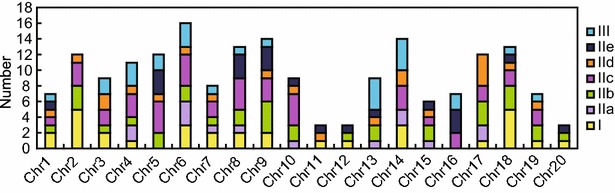
The distribution of WRKY genes on the 20 chromosomes of soybean

In silico mapping revealed that the WRKY genes were distributed over all 20 soybean chromosomes. Chromosome 7 harbored the highest number of *GmWRKY* genes (16, 8.51 %), while chromosome 11, 12 and 20 harbored only three (1.60 %). The largest WRKY product was encoded by *GmWRKY46* (1355 residues), and the shortest was GmWRKY131 (68 residues) (Fig. 1; Table 1).

### Classification of WRKY genes in soybean

As described previously, WRKY family is typically categorized into three main groups defined by the number of WRKY domains present and the configuration of their zinc finger (Rushton et al. 2010). The 188 soybean WRKY genes were also categorized into the three main groups (Additional file 3: Fig. S1). The group I members numbered 32 (GmWRKY65 and -72 harbored a single N-terminal WRKY domain); there were 130 sequences assigned to group II, sub-divided into subgroup IIa (14 members), IIb (33 members), IIc (42 members), IId (21 members) and IIe (20 members); the remaining 26 sequences belonged to group III (Table 1; Fig. 1). In *Arabidopsis* and poplar, group I houses the largest number of WRKYs, while in rice, group III is the largest (He et al. 2012). However, the largest group in soybean is group II, implying that this group had experienced more gene duplications during the evolutionary course.

Although most of the sequences harbored the well conserved WRKYGQK motif, variants were present in 24 of the sequences: WRKYGKK in 11, WRKYGEK in three, WKKYGQK in two, and WRKYGKR, WRKYEDK, WIKYGQK and WHQYGLK each in one. Strikingly, A WRKYGQK-like stretch was lacking in GmWRKY20, -49, -105 and -130, while the group I members WRKY65 and -72 had both lost their N terminal WRKYGQK-like stretch (Table 1; Additional file 3: Fig. S1). The largest number of variants belonged to group IIc, 11 out of 24. The WRKYGQK sequence was highly conserved in subgroups IIb, IId and IIe, as well as in the C terminal WRKY domain of group I members (Table 1; Additional file 3: Fig. S1). This is consistent with the implication that this group experienced more gene duplications. There was also some variation in the zinc finger motif (including its complete absence) in 11 of the sequences (WRKY6, -42, -52, -65, -72, -79, -94, -106, -112, -139 and -165) (Additional file 3: Fig. S1).

### Gene duplication of soybean WRKY genes

**Fig. 2:**
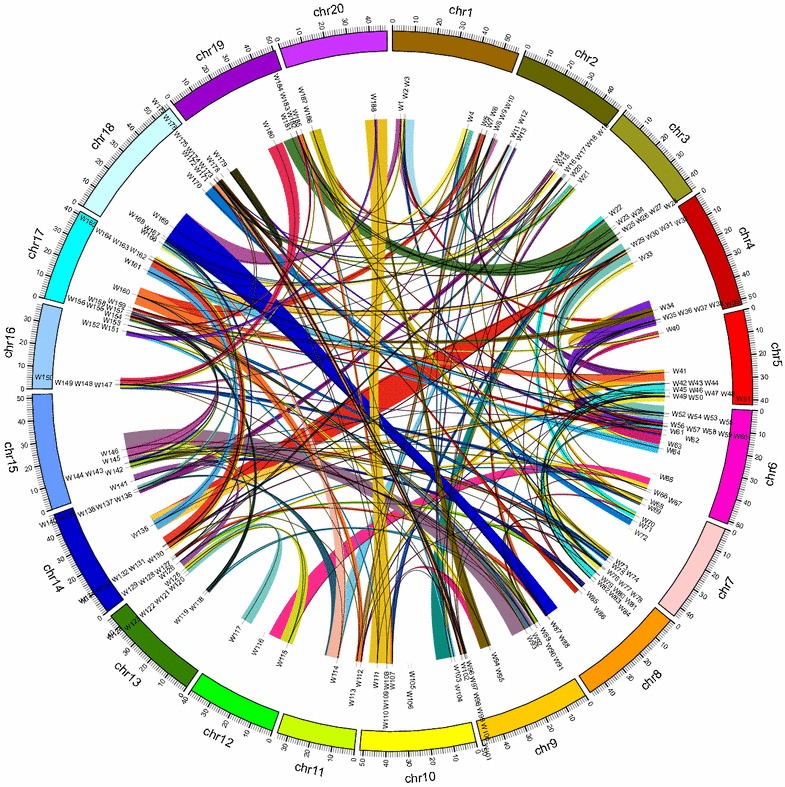
Chromosomal location of the soybean WRKY genes. The illustrated genome-wide chromosome organization caused by whole genome duplication events is accomplished using the Circos software based on the duplication coordinates defined in the current genome assembly v2.0. Segmental duplicated blocks are color coded. Paralogous pairs are connected with lines

Duplication events contribute not only to functional redundancy, but also generate functional novelty (Moore and Purugganan 2005). The modern soybean genome has undergone two whole genome duplication (WGD) events, the first, associated with the evolution of the legume clade occurred ~59 million years ago (Lavin et al. 2005), while the second, which was responsible for the creation of the *Glycine* genus, occurred ~13 million years ago (Schmutz et al. 2010). To investigate whether the expansion of *GmWRKY* genes had primarily happened during both WGD events, we mapped the *GmWRKYs* to the duplicated blocks (Fig. 2). Consistent with previous study (Schmutz et al. 2010), the blocks between chromosomes involved more than just two chromosomes. Of 188 *GmWRKYs*, 180 (95.7 %) genes were located in the blocks (the exceptions were *GmWRKY46*, -*63*, -*65*, -*72*, -*94*, -*105*, -*106*, and -*139*) (Fig. 2), indicating that WGD was the primary reason for the expansion of *GmWRKYs* (Fig. 2).

Besides WGD, tandem duplication event is the other approach for gene expansion. Precise mapping analysis showed the presence of 14 adjacent genes possibly due to tandem duplication (Fig. 2; Additional file 4: Fig. S2a). These 14 WRKY genes were localized in 6 distinct tandem duplicate gene clusters, with four clusters containing two tandem genes (*GmWRKY120/123*, *GmWRKY121/122*, *GmWRKY131/132* and *GmWRKY151/152*) and two clusters possessing three ones (*GmWRKY108/109/110* and *GmWRKY136/137/138*). All the 14 tandem duplicated WRKY genes were mapped onto the duplicated blocks, implying that local duplications occurred earlier than the WGD.

### Gene structure and conserved motifs of *GmWRKYs*

Gene structural diversity may reflect the evolution of multigene families (Hu et al. 2010). In order to look into the structural diversity of *GmWRKY* genes, we first constructed a phylogenetic tree based on the full-length GmWRKY polypeptide sequences, and they were also categorized into seven subfamiles as above (Additional file 4: Fig. S2a). From the tree, we could find that each clade consists of two to four genes, which well matched the two WGD events and confirmed that the expansion of GmWRKY happened during both WGD events. We then analyzed the exon–intron organization in the coding sequences of each soybean WRKY genes HD-ZIP genes (Additional file 4: Fig. S2b). Previous study showed that most *Populus* WRKY genes contain two to four introns (He et al. 2012). Similarly, the majority of soybean WRKY members harbored two to four introns. For instance, over 60 % members of subgroups IIc (26/42), IId (17/21), IIe (14/20) and III (23/26) harbored two introns; over 60 % group I members (17/32) harbored four; most members in group IIa harbored three (7/14) or four (5/14) (Additional file 4: Fig. S2b). In contrast, the gene structure appeared to be more variable in groups IIb, the number of introns in this group varied from one to six (Additional file 4: Fig. S2b). In *Populus*, although there were only eight members in group IIb, the numbers of introns varied from three to six (He et al. 2012). These results indicated that WRKY genes in different species were relatively conserved during the evolution. Furthermore, genes shared similar exon–intron organization within the same subgroup, while they were strikingly distinct in the gene structure among different groups, suggesting that they were not only conserved, but diverged during the evolution.

To better understand the conservation and diversification of WRKY genes in soybean, putative motifs of GmWRKYs were predicted using MEME software and finally 16 distinct motifs were identified (Additional file 4: Fig. S2c). As expected, most of the closely related members in the phylogenetic tree shared common motif compositions, suggesting that the WRKY proteins within the same subfamily might be of similar functions. However, like putative motifs predicted in ZmWRKYs (Gao et al. 2014), the biological significance of most of the putative motifs in GmWRKYs was also unclear because they did not have homologs when searching against Pfam (http://pfam.sanger.ac.uk/search) and SMART (Simple Modular Architecture Research Tool) databases. The same phenomenon also existed in *Populus* NAC and HD-ZIP proteins (Hu et al. 2010, 2012). According to previous study, WRKY proteins harbor typical WRKY domains and zinc-finger motifs (Eulgem et al. 2000; Rushton et al. 2010). Here, motif 1, 2 and 9 comprised the WRKY domain, motif 3 and 10 were the partial zinc-finger motifs followed motif 2 and 9 (Additional file 5: Table S3). The product size of the group I and subgroup IIb genes was larger than that of members of the other groups (Table 1), consistent with their harboring a greater number of motifs (Additional file 4: Fig. S2c). In contrast, although subgroup IIc possessed the largest number of members, they harbored the least number of motifs (one to three). Even though the C-terminal regions of GmWRKYs were highly divergent, we could also identify several conserved motifs which were present in GmWRKYs from specific subgroups, for example, motifs 3, 5 and 6 in group I, motif 14 in subgroup IIb, and motif 13 in subgroups IIa and IIb (Additional file 4: Fig. S2c). Whether these motifs play functional roles remained to be further elucidated.

### Expression profiles of *GmWRKYs* in response to salt stress

**Fig. 3:**
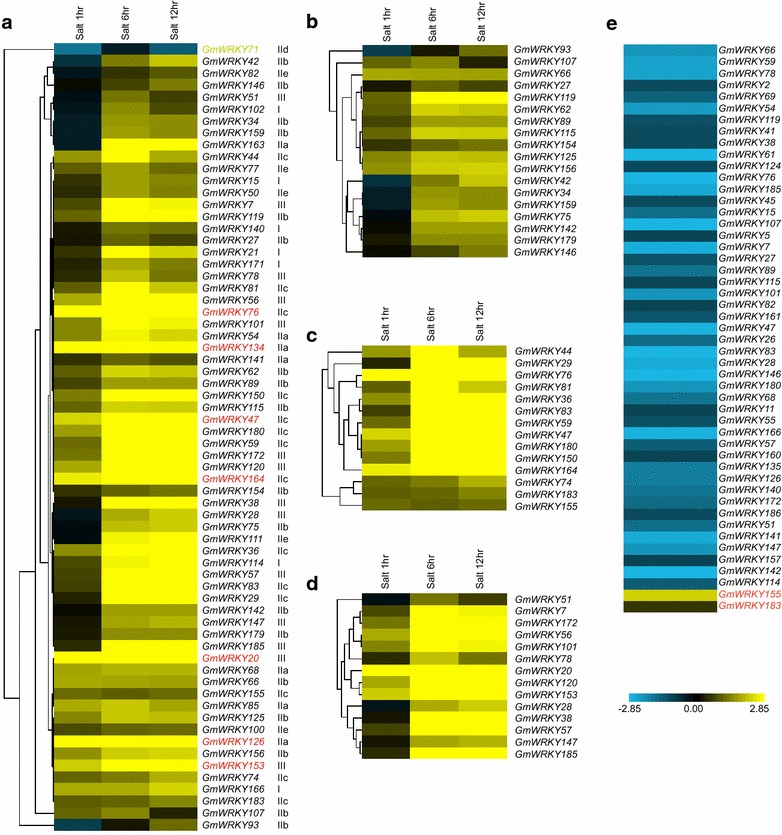
The transcription response of the WRKY genes in response to salt stress. Transcript abundance levels have been normalized and hierarchical clustered. *Blue colored* blocks indicate a decreased and *yellow ones* an increased level of transcription relative to the control. **a** The set of 66 genes transcriptionally altered in soybean root by the stress. Genes with remarkable changed expressions are labeled in *red*. **b**–**d** The transcription profiles of genes belonging to subgroups **b** IIb and **c** IIc and to **d** group III. *hr* number of hours of exposure to the stress. **e** The set of 49 genes transcriptionally altered in the aerial part of soybean by the stress

The WRKY gene family is heavily implicated in the plant response to abiotic stress (Chen et al. 2012), as indicated by a number of microarray-based transcriptomic data sets. Several studies have reported the influence of abiotic stress on WRKY genes based on these data sets (Dou et al. 2014; Satapathy et al. 2014; Wei et al. 2012). In soybean (*cv*. Kefeng No. 1), the response of a set of 64 WRKY genes following the plant’s exposure to salt stress has been described (Zhou et al. 2008), but this number represents only about one-third of the total WRKY genes. In order to give insight to the function of GmWRKYs in plant response to salt tolerance, we analyzed the soybean (*cv*. Williams 82) gene expression profiles under salt stress (Belamkar et al. 2014). Finally, 66 of the 188 *GmWRKY* genes were transcriptionally regulated under salt stress (Fig. 3a; Additional file 6: Table S4). 65 genes were up-regulated, with only *WRKY71* being down-regulated (Fig. 3a). The response of WRKY was typically quite rapid (Eulgem et al. 2000), most notably in the case of *GmWRKY20*, -*47*, -*76*, -*126*, -*134*, -*153*, -*164*, which responded by an at least five fold rise in transcript abundance after a 1 h exposure to the stress. In some cases, the response was transient: some examples were the genes *WRKY44*, -*51*, -*54*, -*78*, -*81*, -*85*, -*102* and -*107*, for which transcript abundance peaked after a 6 h exposure and then fell away (Fig. 3a; Additional file 6: Table S4). The most responsive gene (*WRKY134*) belonged to subgroup IIa, which was increased to ~226 fold after a 6 h exposure (Fig. 3a; Additional file 6: Table S4). By contrast, the expression of *GmWRKY71*, a member of group IId was down-regulated in response to salt stress (Fig. 3a).

**Fig. 4:**
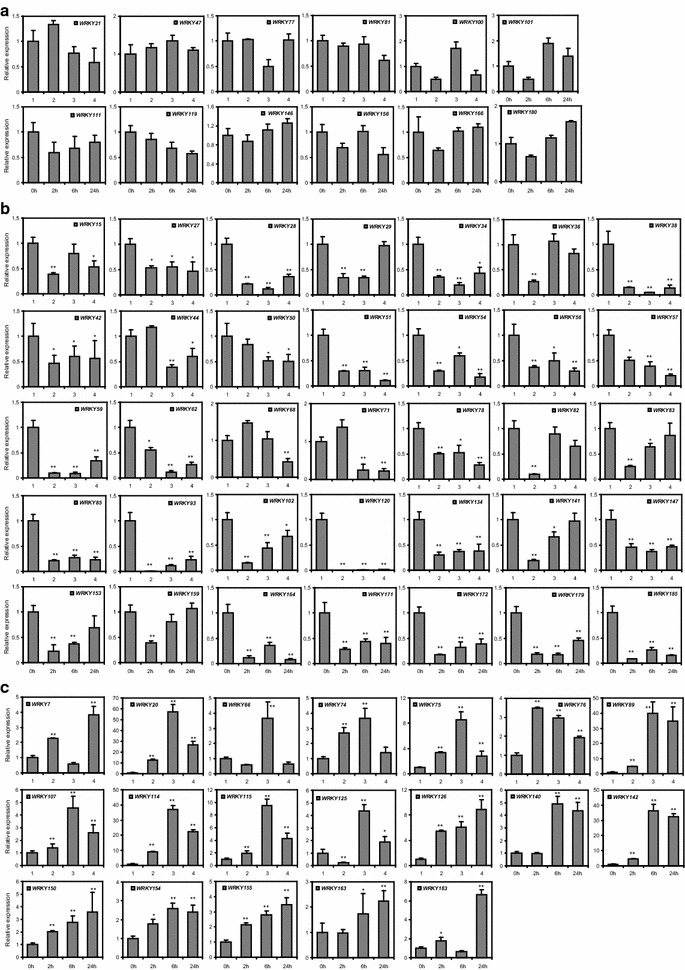
RT-qPCR-based transcription profiling of 66 WRKY genes. **a** Unaffected WRKY genes. **b** Salt-inhibited WRKY genes. **c** Salt-inducible WRKY genes. *Error bars* represent SD (n = 3). (*t* test, *P < 0.05; **P < 0.01)

In soybean, a notable number of responsive genes belonged to subgroup IIb (18 of 33), although their level of induction by salt treatment was only modest (Fig. 3b). In addition, most of subgroup IIc (14/42) and group III (14/26) members were significantly induced and these genes tended to be dramatically up-regulated after a 6 h exposure (Fig. 3c, d). In *Arabidopsis* root, the 18 salt induced members belong to group I (4/18), II (11/18) and III (3/18), respectively (Jiang and Deyholos 2006). These data indicated that either in soybean or *Arabidopsis*, group II members made major contribution in salt response, suggesting that WRKY functions in response to salt stress in different organisms appeared to be conserved during evolution.

The material used in the above RNA-seq was soybean root which was not able to represent other parts. We then analyzed the other RNA-seq data which were derived from the aerial part of soybean plants (*cv*. SuiNong 28) (Wei et al. 2015). A total of 49 *GmWRKY* genes were transcriptionally regulated under salt stress (Fig. 3e; Additional file 7: Table S5). 47 genes were down-regulated, with only two (*WRKY155* and *WRKY183*) being up-regulated (Fig. 3e). These results were quite different with the above RNA-seq analysis, indicating that *GmWRKY* genes showed distinct response profiles in different tissues.

### Investigation of *Gm*WRKY gene expressions by RT-qPCR

The transcriptional profiles we analyzed above could provide clues for revealing the function of GmWRKYs in plant response to salt tolerance. However, the material used in RNA-seq was soybean root or aerial part which was not able to represent the entire plant. To shed light on the expression profiles of *GmWRKY* genes, 2 weeks old soybean seedlings (*cv*. Williams 82) were exposed to 200 mM NaCl for 0, 2, 6 or 24 h, respectively, and then the total RNA of the whole plant was isolated used for RT-qPCR analysis. 66 *GmWRKY* genes were tested and exhibited distinct expression patterns in response to salt stress, of which 12 showed no significant change (Fig. 4a), 35 were decreased (Fig. 4b), while 19 were induced (Fig. 4c). *GmWRKY38*, -*120* and -*185* were substantially decreased, especially *GmWRKY120*. In contrast, *GmWRKY20*, -*89*, -*114* and -*142* were remarkably induced. These expression patterns were different with the above RNA-seq analysis, indicating that *GmWRKY* genes showed distinct response profiles in the whole plants compared to different tissues. The response of WRKY to abiotic stresses was generally rapid and transient (Eulgem et al. 2000). Likely, most of the *GmWRKY* genes responded rapidly, their expressions were decreased (31/35) or induced (16/19) after only a 2 h exposure (Fig. 4b, c). In addition, the response of *GmWRKYs* was transient, such as *GmWRKY36*, -82, -*83*, -*141*, -*153*, -*159* and -*66* (Fig. 4b, c).

In tomato and cucumber, most *WRKYs* are up-regulated by salt stress (Huang et al. 2012; Ling et al. 2011). In contrast, the majority of *Brachypodium distachyonWRKYs* are down-regulated by the stress (Wen et al. 2014). In soybean, most *WRKYs* were up-regulated in root, while down-regulated in the aerial part by the stress (Fig. 4). Species differences presumably reflected a major degree of functional divergence in the WRKY gene family.

## Conclusion

The present study has taken a genome-wide view of the soybean WRKY gene family, and characterized their transcriptional response to salt stress. An analysis of their phylogeny, chromosomal location, gene structure and content of conserved motifs has allowed the genes to be classified into the standard set of groups. The expansion in copy number of the *GmWRKYs* has occurred largely as a result of the two well recognized ancient whole genome duplication events. To date, only three *GmWRKY* genes have been functionally investigated (Jiang and Deyholos 2009), leaving unknown the function of the remaining more than 180. The responsiveness to salt stress of about one-third of the *GmWRKY* complement confirms the potential of gene manipulation within this gene family as means of improving the salt tolerance of important crop species.

## Additional files

10.1186/s40064-016-2042-7 Primers used in this study.
10.1186/s40064-016-2042-7 A comparison between the WRKY genes identified in the current study and those described previously (Bencke-Malato et al. 2014).
10.1186/s40064-016-2042-7 Multiple alignment of the conserved WRKY domain sequences.
10.1186/s40064-016-2042-7 The phylogeny, gene structure and conserved motifs of the soybean WRKY gene family. (**a**) A multiple sequence alignment of 188 full length polypeptide sequences. The seven groups/subgroups (I, IIa-e and III) are depicted by different colors. (**b**) Exon–intron structures. (**c**) The 16 conserved motifs as identified by MEME software. Detailed sequence information given in Additional file 4: Table S3.
10.1186/s40064-016-2042-7 Conserved WRKY motif sequences as predicted by MEME software.
10.1186/s40064-016-2042-7 Normalized transcript levels of 66 GmWRKY genes in root under salinity stress conditions.
10.1186/s40064-016-2042-7 Normalized transcript levels of 49 GmWRKY genes in aerial part under salinity stress conditions.

## Abbreviations

NCBI: National Center for Biotechnology Information
PlantTFDB: Plant Transcription Factor Database
SoyDB: Database of Soybean Transcription Factors
SoyTFKB: Soybean Transcription Factor Knowledge Base
SMART: Simple Modular Architecture Research Tool
GSDS: Gene Structure Display Server
MEME: Multiple Em for Motif Elicitation
