# Supplementary material for: Genome-wide identification of soybean WRKY transcription factors in response to salt stress
Source: Springerplus. 2016 Jun 29;5(1):920. doi: 10.1186/s40064-016-2647-x (PMC4927560; doi:10.1186/s40064-016-2647-x)
Supplement: Supplementary file 1 — 10.1186/s40064-016-2042-7 Primers used in this study. [file 40064_2016_2647_MOESM1_ESM.doc]

**Table S1.** Primers used in this study.

| **Primer Name** | **Sequence (5’-3’)** |
| --- | --- |
| GmWRKY7-real-F | AGTGAAGATCGAGAACGGCG |
| GmWRKY7-real-R | AAGCACCCCTTTGTTTTGCG |
| GmWRKY15-real-F | ACACCTCTGCAAGTGCAAACT |
| GmWRKY15-real-R | TGGTGGTTTGTTGTTGTCTGTG |
| GmWRKY20-real-F | TGCATGCAACTAATACAATCGCA |
| GmWRKY20-real-R | GCATGTGTGGTGGCTCAAAT |
| GmWRKY21-real-F | ACAAACCACGTGCCTCTCTT |
| GmWRKY21-real-R | TTGCTTTGCAGCTTCACTGG |
| GmWRKY27-real-F | TCTTAAGCTCCGCCCACTTC |
| GmWRKY27-real-R | TCCATGGCGATCATGAGTGT |
| GmWRKY28-real-F | ACATACATGCACGCAAGCTAA |
| GmWRKY28-real-R | TGAAAATCCCCTCAGGGTTCTG |
| GmWRKY29-real-F | AGCAGAGACGTTGGCAATGA |
| GmWRKY29-real-R | AGTTCCTTGGATTGGGGCTG |
| GmWRKY34-real-F | TTGGCCACAAAGGAAGGGAC |
| GmWRKY34-real-R | GGTGCCACATCTTGCCCTAA |
| GmWRKY36-real-F | GCAACCTTCAGCAACACCAA |
| GmWRKY36-real-R | AACCTCAAGCTGCGATCTGG |
| GmWRKY38-real-F | GTTGCCGAGCAACCAAACAA |
| GmWRKY38-real-R | GGCACATCATTTGCGTCAGG |
| GmWRKY42-real-F | TATCGTTGCACTGTAGCCCC |
| GmWRKY42-real-R | GCAGAAGAGGGAGTGATGGT |
| GmWRKY44-real-F | CACCCTCACCACCAATATCACA |
| GmWRKY44-real-R | TCCGGAGGAAGAAACTGGTTG |
| GmWRKY47-real-F | CTCACGGATTCAGCACTGGT |
| GmWRKY47-real-R | TCCTTGGACCCACTCCTCTT |
| GmWRKY50-real-F | CATCCCCACCCAACTCGAAG |
| GmWRKY50-real-R | TCCATGCTTTGTTGAACGCT |
| GmWRKY51-real-F | CTTGGGCCCTGGAGAACGAG |
| GmWRKY51-real-R | GGATTCCGAATGTGGCTTGCT |
| GmWRKY54-real-F | AACTTACGAAGGGGAGCACA |
| GmWRKY54-real-R | TCATGGCTGAGGGTGAAACT |
| GmWRKY56-real-F | TAGGCCACTTTTCTGTGTCTCC |
| GmWRKY56-real-R | TGATGAGTTTGGAGCTGCTGA |
| GmWRKY57-real-F | GCAACCAAACAAGTGCAACG |
| GmWRKY57-real-R | GGCACATTTGATTCAGGACC |
| GmWRKY59-real-F | TGTTCAGGTGAAGGATGCGA |
| GmWRKY59-real-R | TAGGCAGTAGACGGGGTCTG |
| GmWRKY62-real-F | AAGCAGCTGGTGAGAGTTGG |
| GmWRKY62-real-R | TGGAGTGTCACATCTTGCCC |
| GmWRKY66-real-F | CTTCCTCCACAAGCCAATTGTT |
| GmWRKY66-real-R | CTTTGGTATTGCTAGCTGTGGC |
| GmWRKY68-real-F | GGGAGCACAATCATCCTCAGT |
| GmWRKY68-real-R | GAAGTGCTGAGAGATGCGGTA |
| GmWRKY71-real-F | TTCCTCATGGGCCAACAACA |
| GmWRKY71-real-R | CTATGGGTTTGGTCCTGGGC |
| GmWRKY74-real-F | TCTGTGGCATTTTCCGACGA |
| GmWRKY74-real-R | GCCAGCAAAGTTGTTGGGAG |
| GmWRKY75-real-F | AACGCACTGAGAACACGGAA |
| GmWRKY75-real-R | CGGTCGTACCACTCGGAAC |
| GmWRKY76-real-F | AAAGGCCAAGCCTGAAAGGT |
| GmWRKY76-real-R | TCTCCACCGGTACCCATCAT |
| GmWRKY77-real-F | ACCCAGTTTTGCACCCTCTT |
| GmWRKY77-real-R | GCAGGGCTGCTACTTTCTGT |
| GmWRKY78-real-F | AGCAATGCATCAACTTCCGAG |
| GmWRKY78-real-R | TGGTGATCCTTGATGGCCCC |
| GmWRKY81-real-F | CACCACTGCTACCATCGCTC |
| GmWRKY81-real-R | GGCCGTATTCTGGAGGAAGA |
| GmWRKY82-real-F | GGTGCTCGAAAGCCCCATTA |
| GmWRKY82-real-R | CCTGCCTGAATACCACCGAG |
| GmWRKY83-real-F | ATCTTCCCCTGAGATTGCAC |
| GmWRKY83-real-R | CCGTGAGTGGCATCATTGAA |
| GmWRKY85-real-F | AGAAGCTGTTCCTAAGGTGGTA |
| GmWRKY85-real-R | CTTGTTTTCTGCACTCACCCG |
| GmWRKY89-real-F | CCTCCCACACCCGACAATAC |
| GmWRKY89-real-R | GTTGGGTGGCGGAGTATGAG |
| GmWRKY93-real-F | TGCAGCAGCATCAACTATCAG |
| GmWRKY93-real-R | ACTGCCGAGTGTGTAGCATT |
| GmWRKY100-real-F | ATCGTCGGTGTCTTCCTCATCTT |
| GmWRKY100-real-R | GCCTCAAAAGGGTACGCAGAA |
| GmWRKY101-real-F | GATGATGGATCAGCCACCCC |
| GmWRKY101-real-R | CCTCAGACTTGGGAGAGTCC |
| GmWRKY102-real-F | TTGAGCAAACACCGCAAACC |
| GmWRKY102-real-R | ACAGATCTGCTTGCTGAGACT |
| GmWRKY107-real-F | GCAGTGTCAGAAGATGGAGAC |
| GmWRKY107-real-R | ATGGTGCAACTTGAGCGTCT |
| GmWRKY111-real-F | AACAACACTAGTGCTCCGGG |
| GmWRKY111-real-R | TCTGGCACAAAATGAGGCCA |
| GmWRKY114-real-F | TCCAACGTTCTGCCATCTCC |
| GmWRKY114-real-R | AGGGTGTGTTTGTGTTGGGA |
| GmWRKY115-real-F | TAGACGTGGTGGGCTATCCA |
| GmWRKY115-real-R | ACTCGGGGTTGTGGTTGTTT |
| GmWRKY119-real-F | TGGAGAAACCAGCGTTACCC |
| GmWRKY119-real-R | TTCTGATCGCGCTCTTACGG |
| GmWRKY120-real-F | AAGACGAAGTGGGAGCTACA |
| GmWRKY120-real-R | AGTGCATCTGTAGTAGTTCCTG |
| GmWRKY125-real-F | TCCAAGAAGAATGGGGGTGC |
| GmWRKY125-real-R | CCCCTTGATCCGAGGGACTA |
| GmWRKY126-real-F | GCTCCAACCGTTGTATGACT |
| GmWRKY126-real-R | TGCGGGACTTCTGGTGATTC |
| GmWRKY134-real-F | ACGCAAGAGAAAAGCCGAGA |
| GmWRKY134-real-R | TGAATCCTCGGTGATGGAGC |
| GmWRKY140-real-F | GCAAGTGCAAACTCCTTCACC |
| GmWRKY140-real-R | GGCTTGTTGTTGTCCAAGGG |
| GmWRKY141-real-F | TTGAACCTCTCACCTTGCTCA |
| GmWRKY141-real-R | TGGGAAGTTCTTCGTGAACCC |
| GmWRKY142-real-F | GATTCCCCGTCAACCTCAGC |
| GmWRKY142-real-R | GTTGTGGTCGTGAGAGGGTG |
| GmWRKY146-real-F | CACCAGCAGCAAGACCTTTG |
| GmWRKY146-real-R | TGGGGAAATAGTGGATAGGGGT |
| GmWRKY147-real-F | AAGGGAAGGAGCTAGCAAAGC |
| GmWRKY147-real-R | GCTGCCATTAATGTTGGCCTT |
| GmWRKY150-real-F | TGATCATGACAACGGAGGCG |
| GmWRKY150-real-R | CCGGAACCACCTAACTCATCA |
| GmWRKY153-real-F | CAACCAAACAAGAGTGCCCC |
| GmWRKY153-real-R | AAGGAGGTTTCTGGTGGGGA |
| GmWRKY154-real-F | TCTTCCGGTGAAAGGACTCG |
| GmWRKY154-real-R | CGTAGGGTTGGAAGGGTTCAA |
| GmWRKY155-real-F | CATCAGCGAGGTTTTGTGCC |
| GmWRKY155-real-R | CTCCTCATTCCCCATCTGCG |
| GmWRKY156-real-F | CCTGAACCAATTGGGCTTGC |
| GmWRKY156-real-R | GTTTCTTCTGCTGCATCGCC |
| GmWRKY159-real-F | GTGTCGGAGAAAGGTTGTGC |
| GmWRKY159-real-R | GGTGGTAGAAGAAAACGGCA |
| GmWRKY163-real-F | ACACAAAGCTTGAGACCCATCTT |
| GmWRKY163-real-R | GTCTCGGTCTCTTGTTTGTCTGA |
| GmWRKY164-real-F | TTGAAGGGTCTTCTAGCAAAGAT |
| GmWRKY164-real-R | TTCCTCCATCTGTAGCCATCA |
| GmWRKY166-real-F | CACATTTCAACCCCAACATGC |
| GmWRKY166-real-R | CATCTCCAGGCTCCTCTTTGG |
| GmWRKY171-real-F | TTGGCCAGAGCTTCAACTGG |
| GmWRKY171-real-R | TTCTTAAGCGGTTCCTGCACT |
| GmWRKY172-real-F | TGTCATCAATGGGAGTTGTGAA |
| GmWRKY172-real-R | CAAGAACGAGAGGGAGTTGGT |
| GmWRKY179-real-F | AGGCTCTTCAACATCTCAGCC |
| GmWRKY179-real-R | GGGAGTAAATGTGAGGAAGGGG |
| GmWRKY180-real-F | TCCAATTCCTCAAGCTACCCA |
| GmWRKY180-real-R | ATCACCACCACCTTGTCTCTT |
| GmWRKY183-real-F | ATGGAGCTATTGGGTGTGCAA |
| GmWRKY183-real-R | CGGTTGCTGGTTGTTGGTTAC |
| GmWRKY185-real-F | AGACCACCAGTGGCAACATC |
| GmWRKY185-real-R | GTCCATCTGGGCATGGTCTT |
| GmELF1b-real-F | GTTGAAAAGCCAGGGGACA |
| GmELF1b-real-R | TCTTACCCCTTGAGCGTGG |
