# Supplementary material for: Genome-wide identification of soybean WRKY transcription factors in response to salt stress
Source: Springerplus. 2016 Jun 29;5(1):920. doi: 10.1186/s40064-016-2647-x (PMC4927560; doi:10.1186/s40064-016-2647-x)
Supplement: Supplementary file 2 — 10.1186/s40064-016-2042-7 A comparison between the WRKY genes identified in the current study and those described previously (Bencke-Malato et al. 2014). [file 40064_2016_2647_MOESM2_ESM.doc]

**Table S2.** A comparison of *GmWRKY* genes identified in current study with those of previous study ([Bencke-Malato et al, 2014).](#_ENREF_1)

| Gene Name (Current study) | Gene ID ­­(V2.0) | Gene Name (Bencke-Malato  et al. 2014) | Gene ID ­­(V1.1) |
| --- | --- | --- | --- |
| *GmWRKY1* | Glyma.01G043300 | *GmWRKY3* | Glyma01g05050 |
| *GmWRKY2* | Glyma.01G053800 | *GmWRKY9* | Glyma01g06550 |
| *GmWRKY3* | Glyma.01G056800 | *GmWRKY28* | Glyma01g06870 |
| *GmWRKY4* | Glyma.01G128100 | *GmWRKY5* | Glyma01g31921 |
| *GmWRKY5* | Glyma.01G189100 | *GmWRKY35* | Glyma01g39600 |
| *GmWRKY6* | Glyma.01G222300 | *GmWRKY65* | Glyma01g43130 |
| *GmWRKY7* | Glyma.01G224800 | *GmWRKY12* | Glyma01g43420 |
| *GmWRKY8* | Glyma.02G007500 | *GmWRKY66* | Glyma02g01031 |
| *GmWRKY9* | Glyma.02G010900 | *GmWRKY67* | Glyma02g01420 |
| *GmWRKY10* | Glyma.02G020300 | *GmWRKY68* | Glyma02g02430 |
| *GmWRKY11* | Glyma.02G112100 | *GmWRKY69* | Glyma02g12490 |
| *GmWRKY12* | Glyma.02G115200 | *GmWRKY32* | Glyma02g12830 |
| *GmWRKY13* | Glyma.02G141000 | *GmWRKY22* | Glyma02g15920 |
| *GmWRKY14* | Glyma.02G203800 | *GmWRKY70* | Glyma02g36510 |
| *GmWRKY15* | Glyma.02G232600 | *GmWRKY39* | Glyma02g39870 |
| *GmWRKY16* | Glyma.02G285900 | *GmWRKY71* | Glyma02g45530 |
| *GmWRKY17* | Glyma.02G293400 | *GmWRKY72* | Glyma02g46280 |
| *GmWRKY18* | Glyma.02G297400 | *GmWRKY73* | Glyma02g46690 |
| *GmWRKY19* | Glyma.02G306300 | *GmWRKY74* | Glyma02g47650 |
| *GmWRKY20* | Glyma.03G002300 | *GmWRKY75* | Glyma03g00460 |
| *GmWRKY21* | Glyma.03G042700 | *GmWRKY76* | Glyma03g05220 |
| *GmWRKY22* | Glyma.03G109100 | *GmWRKY77* | Glyma03g25770 |
| *GmWRKY23* | Glyma.03G159700 | *GmWRKY15* | Glyma03g31630 |
| *GmWRKY24* | Glyma.03G176600 | *GmWRKY29* | Glyma03g33376 |
| *GmWRKY25* | Glyma.03G220100 | *GmWRKY41* | Glyma03g37870 |
| *GmWRKY26* | Glyma.03G220800 | *GmWRKY51* | Glyma03g37940 |
| *GmWRKY27* | Glyma.03G224700 | *GmWRKY78* | Glyma03g38360 |
| *GmWRKY28* | Glyma.03G256700 | *GmWRKY43* | Glyma03g41750 |
| *GmWRKY29* | Glyma.04G054200 | *GmWRKY79* | Glyma04g05700 |
| *GmWRKY30* | Glyma.04G061300 | *GmWRKY80* | Glyma04g06470 |
| *GmWRKY31* | Glyma.04G061400 | *GmWRKY81* | Glyma04g06495 |
| *GmWRKY32* | Glyma.04G076200 | *GmWRKY50* | Glyma04g08060 |
| *GmWRKY33* | Glyma.04G115500 | *GmWRKY82* | Glyma04g12830 |
| *GmWRKY34* | Glyma.04G173500 | *GmWRKY83* | Glyma04g34220 |
| *GmWRKY35* | Glyma.04G218400 | *GmWRKY45* | Glyma04g39621 |
| *GmWRKY36* | Glyma.04G218700 | *GmWRKY21* | Glyma04g39650 |
| *GmWRKY37* | Glyma.04G223200 | *GmWRKY84* | Glyma04g40121 |
| *GmWRKY38* | Glyma.04G223300 | *GmWRKY58* | Glyma04g40130 |
| *GmWRKY39* | Glyma.04G238300 | *GmWRKY85* | Glyma04g41701 |
| *GmWRKY40* | Glyma.05G029000 | *GmWRKY86* | Glyma05g01285 |
| *GmWRKY41* | Glyma.05G096500 | *GmWRKY11* | Glyma05g20710 |
| *GmWRKY42* | Glyma.05G123000 | *GmWRKY87* | Glyma05g25270 |
| *GmWRKY43* | Glyma.05G123600 | *GmWRKY88* | Glyma05g25331 |
| *GmWRKY44* | Glyma.05G127600 | *GmWRKY89* | Glyma05g25770 |
| *GmWRKY45* | Glyma.05G160800 | *GmWRKY90* | Glyma05g29310 |
| *GmWRKY46* | Glyma.05G165800 | *GmWRKY91* | Glyma05g29921 |
| *GmWRKY47* | Glyma.05G184500 | *GmWRKY92* | Glyma05g31800 |
| *GmWRKY48* | Glyma.05G185400 | *GmWRKY93* | Glyma05g31910 |
| *GmWRKY49* | Glyma.05G203900 |  |  |
| *GmWRKY50* | Glyma.05G211900 | *GmWRKY94* | Glyma05g37390 |
| *GmWRKY51* | Glyma.05G215900 | *GmWRKY46* | Glyma05g36970 |
| *GmWRKY52* | Glyma.06G054500 | *GmWRKY95* | Glyma06g05721 |
| *GmWRKY53* | Glyma.06G061900 |  |  |
| *GmWRKY54* | Glyma06g06530 | *GmWRKY17* | Glyma06g06530 |
| *GmWRKY55* | Glyma.06G077400 | *GmWRKY37* | Glyma06g08120 |
| *GmWRKY56* | Glyma.06G125600 | *GmWRKY96* | Glyma06g13090 |
| *GmWRKY57* | Glyma.06G142000 | *GmWRKY97* | Glyma06g14720 |
| *GmWRKY58* | Glyma.06G142100 | *GmWRKY98* | Glyma06g14731 |
| *GmWRKY59* | Glyma.06G147100 | *GmWRKY61* | Glyma06g15220 |
| *GmWRKY60* | Glyma.06G147500 | *GmWRKY99* | Glyma06g15260 |
| *GmWRKY61* | Glyma.06G168400 | *GmWRKY100* | Glyma06g17690 |
| *GmWRKY62* | Glyma.06G190800 | *GmWRKY101* | Glyma06g20300 |
| *GmWRKY63* | Glyma.06G212900 | *GmWRKY102* | Glyma06g23990 |
| *GmWRKY64* | Glyma.06G219800 | *GmWRKY103* | Glyma06g27440 |
| *GmWRKY65* | Glyma.06G242200 | *GmWRKY104* | Glyma06g37100 |
| *GmWRKY66* | Glyma.06G307700 | *GmWRKY105* | Glyma06g46420 |
| *GmWRKY67* | Glyma.06G320700 | *GmWRKY59* | Glyma06g47880 |
| *GmWRKY68* | Glyma.07G023300 | *GmWRKY106* | Glyma07g02630 |
| *GmWRKY69* | Glyma.07G057400 | *GmWRKY55* | Glyma07g06320 |
| *GmWRKY70* | Glyma.07G116300 | *GmWRKY107* | Glyma07g13611 |
| *GmWRKY71* | Glyma.07G133700 | *GmWRKY108* | Glyma07g16040 |
| *GmWRKY72* | Glyma.07G161100 |  |  |
| *GmWRKY73* | Glyma.07G227200 | *GmWRKY109* | Glyma07g35381 |
| *GmWRKY74* | Glyma.07G238000 | *GmWRKY110* | Glyma07g36640 |
| *GmWRKY75* | Glyma.07G262700 | *GmWRKY34* | Glyma07g39250 |
| *GmWRKY76* | Glyma.08G011300 | *GmWRKY25* | Glyma08g01430 |
| *GmWRKY77* | Glyma.08G018300 | *GmWRKY111* | Glyma08g02160 |
| *GmWRKY78* | Glyma.08G021900 | *GmWRKY20* | Glyma08g02580 |
| *GmWRKY79* | Glyma.08G078100 | *GmWRKY112* | Glyma.08G078100 |
| *GmWRKY80* | Glyma.08G078700 | *GmWRKY113* | Glyma08g08340 |
| *GmWRKY81* | Glyma.08G082400 | *GmWRKY114* | Glyma08g08720 |
| *GmWRKY82* | Glyma.08G118200 | *GmWRKY48* | Glyma08g12460 |
| *GmWRKY83* | Glyma.08G142400 | *GmWRKY6* | Glyma08g15050 |
| *GmWRKY84* | Glyma.08G143400 | *GmWRKY40* | Glyma08g15210 |
| *GmWRKY85* | Glyma.08G218600 | *GmWRKY56* | Glyma08g23380 |
| *GmWRKY86* | Glyma.08G240800 | *GmWRKY4* | Glyma08g26230 |
| *GmWRKY87* | Glyma.08G320200 | *GmWRKY115* | Glyma08g43258 |
| *GmWRKY88* | Glyma.08G325800 | *GmWRKY18* | Glyma08g43770 |
| *GmWRKY89* | Glyma.09G005700 | *GmWRKY23* | Glyma09g00820 |
| *GmWRKY90* | Glyma.09G029800 | *GmWRKY116* | Glyma09g03451 |
| *GmWRKY91* | Glyma.09G034300 | *GmWRKY26* | Glyma09g03900 |
| *GmWRKY92* | Glyma.09G061900 | *GmWRKY47* | Glyma09g06980 |
| *GmWRKY93* | Glyma.09G080000 | *GmWRKY117* | Glyma09g09400 |
| *GmWRKY94* | Glyma.09G127100 | *GmWRKY118* | Glyma.09g127100 |
| *GmWRKY95* | Glyma.09G129100 | *GmWRKY119* | Glyma09g24080 |
| *GmWRKY96* | Glyma.09G240000 | *GmWRKY120* | Glyma09g37470 |
| *GmWRKY97* | Glyma.09G244000 | *GmWRKY121* | Glyma09g37930 |
| *GmWRKY98* | Glyma.09G250500 | *GmWRKY122* | Glyma09g38581 |
| *GmWRKY99* | Glyma.09G254400 | *GmWRKY123* | Glyma09g39000 |
| *GmWRKY100* | Glyma.09G254800 | *GmWRKY124* | Glyma09g39040 |
| *GmWRKY101* | Glyma.09G274000 | *GmWRKY125* | Glyma09g41050 |
| *GmWRKY102* | Glyma.09G280200 | *GmWRKY126* | Glyma09g41670 |
| *GmWRKY103* | Glyma.10G011300 | *GmWRKY54* | Glyma10g01450 |
| *GmWRKY104* | Glyma.10G032900 | *GmWRKY127* | Glyma10g03820 |
| *GmWRKY105* | Glyma.10G111400 | *GmWRKY129* | Glyma10g14610 |
| *GmWRKY106* | Glyma.10G113800 | *GmWRKY128* | Glyma10g13720 |
| *GmWRKY107* | Glyma.10G138300 | *GmWRKY1* | Glyma10g27860 |
| *GmWRKY108* | Glyma.10G171000 |  |  |
| *GmWRKY109* | Glyma.10G171100 | *GmWRKY130* | Glyma10g31396 |
| *GmWRKY110* | Glyma.10G171200 | *GmWRKY131* | Glyma10g31413 |
| *GmWRKY111* | Glyma.10G230200 | *GmWRKY2* | Glyma10g37460 |
| *GmWRKY112* | Glyma.11G021200 | *GmWRKY132* | Glyma11g02361 |
| *GmWRKY113* | Glyma.11G053100 | *GmWRKY14* | Glyma11g05650 |
| *GmWRKY114* | Glyma.11G163300 | *GmWRKY19* | Glyma11g29720 |
| *GmWRKY115* | Glyma.12G097100 | *GmWRKY133* | Glyma12g10350 |
| *GmWRKY116* | Glyma.12G152600 | *GmWRKY44* | Glyma12g23950 |
| *GmWRKY117* | Glyma.12G212300 | *GmWRKY16* | Glyma12g33990 |
| *GmWRKY118* | Glyma.13G102000 | *GmWRKY13* | Glyma13g00380 |
| *GmWRKY119* | Glyma.13G117600 | *GmWRKY134* | Glyma13g17800 |
| *GmWRKY120* | Glyma.13G267400 | *GmWRKY135* | Glyma13g34240 |
| *GmWRKY121* | Glyma.13G267500 | *GmWRKY136* | Glyma13g34251 |
| *GmWRKY122* | Glyma.13G267600 | *GmWRKY137* | Glyma13g34261 |
| *GmWRKY123* | Glyma.13G267700 | *GmWRKY138* | Glyma13g34281 |
| *GmWRKY124* | Glyma.13G289400 | *GmWRKY52* | Glyma13g36540 |
| *GmWRKY125* | Glyma.13G310100 | *GmWRKY36* | Glyma13g38630 |
| *GmWRKY126* | Glyma.13G370100 | *GmWRKY139* | Glyma13g44730 |
| *GmWRKY127* | Glyma.14G006800 | *GmWRKY140* | Glyma14g01010 |
| *GmWRKY128* | Glyma.14G016200 | *GmWRKY7* | Glyma14g01980 |
| *GmWRKY129* | Glyma.14G028900 | *GmWRKY141* | Glyma14g03280 |
| *GmWRKY130* | Glyma.14G085500 |  |  |
| *GmWRKY131* | Glyma.14G100100 |  |  |
| *GmWRKY132* | Glyma14g11440 | *GmWRKY142* | Glyma14g11440 |
| *GmWRKY133* | Glyma.14G102900 | *GmWRKY143* | Glyma14g11920 |
| *GmWRKY134* | Glyma.14G103100 | *GmWRKY144* | Glyma14g11960 |
| *GmWRKY135* | Glyma.14G135400 | *GmWRKY31* | Glyma14g17730 |
| *GmWRKY136* | Glyma.14G185800 | *GmWRKY145* | Glyma14g36430 |
| *GmWRKY137* | Glyma.14G186000 | *GmWRKY146* | Glyma14g36438 |
| *GmWRKY138* | Glyma.14G186100 | *GmWRKY147* | Glyma14g36446 |
| *GmWRKY139* | Glyma.14G199800 | *GmWRKY148* | Glyma14g37960 |
| *GmWRKY140* | Glyma.14G200200 | *GmWRKY49* | Glyma14g38010 |
| *GmWRKY141* | Glyma.15G003300 | *GmWRKY27* | Glyma15g00570 |
| *GmWRKY142* | Glyma.15G110300 | *GmWRKY149* | Glyma15g11680 |
| *GmWRKY143* | Glyma.15G135600 | *GmWRKY150* | Glyma15g14371 |
| *GmWRKY144* | Glyma.15G139000 | *GmWRKY151* | Glyma15g14860 |
| *GmWRKY145* | Glyma.15G168200 | *GmWRKY42* | Glyma15g18250 |
| *GmWRKY146* | Glyma.15G186300 | *GmWRKY152* | Glyma15g20990 |
| *GmWRKY147* | Glyma.16G026400 | *GmWRKY60* | Glyma16g02960 |
| *GmWRKY148* | Glyma.16G031400 | *GmWRKY153* | Glyma16g03480 |
| *GmWRKY149* | Glyma.16G031900 | *GmWRKY154* | Glyma16g03570 |
| *GmWRKY150* | Glyma.16G054400 | *GmWRKY155* | Glyma16g05880 |
| *GmWRKY151* | Glyma.16G176700 | *GmWRKY156* | Glyma.16G176700 |
| *GmWRKY152* | Glyma.16G177000 | *GmWRKY157* | Glyma16g29561 |
| *GmWRKY153* | Glyma.16G219800 | *GmWRKY158* | Glyma16g34590 |
| *GmWRKY154* | Glyma.17G011400 | *GmWRKY159* | Glyma17g01490 |
| *GmWRKY155* | Glyma.17G035400 | *GmWRKY160* | Glyma17g03950 |
| *GmWRKY156* | Glyma.17G042300 | *GmWRKY161* | Glyma17g04710 |
| *GmWRKY157* | Glyma.17G057100 | *GmWRKY33* | Glyma17g06450 |
| *GmWRKY158* | Glyma.17G074000 | *GmWRKY24* | Glyma17g08170 |
| *GmWRKY159* | Glyma.17G097900 | *GmWRKY162* | Glyma17g10630 |
| *GmWRKY160* | Glyma.17G168900 | *GmWRKY163* | Glyma17g18480 |
| *GmWRKY161* | Glyma.17G197500 | *GmWRKY164* | Glyma17g29190 |
| *GmWRKY162* | Glyma.17G222300 | *GmWRKY30* | Glyma17g33891 |
| *GmWRKY163* | Glyma.17G222500 | *GmWRKY63* | Glyma17g33920 |
| *GmWRKY164* | Glyma.17G224800 | *GmWRKY165* | Glyma17g34210 |
| *GmWRKY165* | Glyma.17G239200 | *GmWRKY166* | Glyma17g35750 |
| *GmWRKY166* | Glyma.18G056600 | *GmWRKY62* | Glyma18g06360 |
| *GmWRKY167* | Glyma.18G081200 | *GmWRKY167* | Glyma18g09040 |
| *GmWRKY168* | Glyma.18G092200 | *GmWRKY168* | Glyma18g10324 |
| *GmWRKY169* | Glyma.18G124700 | *GmWRKY169* | Glyma18g16170 |
| *GmWRKY170* | Glyma.18G183100 | *GmWRKY170* | Glyma18g39970 |
| *GmWRKY171* | Glyma.18G208800 | *GmWRKY171* | Glyma18g44030 |
| *GmWRKY172* | Glyma.18G213200 | *GmWRKY57* | Glyma18g44560 |
| *GmWRKY173* | Glyma.18G238200 | *GmWRKY10* | Glyma18g47300 |
| *GmWRKY174* | Glyma.18G238600 | *GmWRKY64* | Glyma18g47350 |
| *GmWRKY175* | Glyma.18G242000 | *GmWRKY172* | Glyma18g47741 |
| *GmWRKY176* | Glyma.18G256500 | *GmWRKY173* | Glyma18g49140 |
| *GmWRKY177* | Glyma.18G263400 | *GmWRKY174* | Glyma18g49830 |
| *GmWRKY178* | Glyma18g48460 | *GmWRKY38* | Glyma18g48460 |
| *GmWRKY179* | Glyma.19G020600 | *GmWRKY175* | Glyma19g02440 |
| *GmWRKY180* | Glyma.19G094100 | *GmWRKY53* | Glyma19g26400 |
| *GmWRKY181* | Glyma.19G177400 | *GmWRKY176* | Glyma19g36100 |
| *GmWRKY182* | Glyma.19G217000 | *GmWRKY8* | Glyma19g40470 |
| *GmWRKY183* | Glyma.19G217800 | *GmWRKY177* | Glyma19g40560 |
| *GmWRKY184* | Glyma.19G221700 | *GmWRKY178* | Glyma19g40950 |
| *GmWRKY185* | Glyma.19G254800 | *GmWRKY179* | Glyma19g44380 |
| *GmWRKY186* | Glyma.20G028000 | *GmWRKY180* | Glyma20g03410 |
| *GmWRKY187* | Glyma.20G030500 | *GmWRKY181* | Glyma20g03820 |
| *GmWRKY188* | Glyma.20G163200 | *GmWRKY182* | Glyma20g30290 |
