# Supplementary material for: Genome-wide identification of soybean WRKY transcription factors in response to salt stress
Source: Springerplus. 2016 Jun 29;5(1):920. doi: 10.1186/s40064-016-2647-x (PMC4927560; doi:10.1186/s40064-016-2647-x)
Supplement: Supplementary file 5 — 10.1186/s40064-016-2042-7 Conserved WRKY motif sequences as predicted by MEME software. [file 40064_2016_2647_MOESM5_ESM.doc]

**Table S3.** Conserved motif sequences of GmWRKY predicted by MEME.

| Motif | Sequence |
| --- | --- |
| 1 | QTKSEVDILDDGYR**WRKYGQK**VVKGNPYPRSYYRCTHAGCNVRKHVERASHDPKIVITTYEGKHNH |
| 2 | VAADRRADDGYN**WRKYGQK**QVKGSEFPRSYYKCTHPNCCVKK |
| 3 | VERSHDGHITEIIYKGTHNHPKPQPNRRYSE |
| 4 | MKKKGEKKIREPRFCF |
| 5 | YFTIPPGLSPTEFLDSPVFLNNMNIFPSPTT |
| 6 | YTGDEADNDEPDAKRWKMENEN |
| 7 | QTHTSNSNFKFVHTTSAPVYFGVLNNNSNPYGSRDNRSDGPSLNHSAYPCPQNMGRILMG |
| 8 | EAFVGQSFNWRNSYGENQQIVKEEEKNYSDFSFQTQTHPPLPM |
| 9 | WQ**WRKYGQK**MIKGNPYPRGYYR |
| 10 | CPARKQVQRCAEDPTMLIVTYE |
| 11 | GTHNHPLPPAAMAMACTTSAAASMLLSGSMTSHHGLMNSNILT |
| 12 | NELEVLQEELGRVKEENQRLREMLDHVCENYNALQMHFMEIMQ |
| 13 | DLVEAMTAAITADPNFTAALAAAITSIIGGG |
| 14 | APMRKARVCVRARCEAPMMNDG |
| 15 | MATISASAPFPTITLDLTHNPN |
| 16 | KKRKMRQKWVVCVPAI |
