# Supplementary material for: Genome-wide identification of soybean WRKY transcription factors in response to salt stress
Source: Springerplus. 2016 Jun 29;5(1):920. doi: 10.1186/s40064-016-2647-x (PMC4927560; doi:10.1186/s40064-016-2647-x)
Supplement: Supplementary file 6 — 10.1186/s40064-016-2042-7 Normalized transcript levels of 66 GmWRKY genes in root under salinity stress conditions. [file 40064_2016_2647_MOESM6_ESM.doc]

**Table S4.** Normalized transcript levels of 66 GmWRKY genes under salinity stress conditions.

| Gene name | 1hr | 6hr | 12hr | group |
| --- | --- | --- | --- | --- |
| *GmWRKY7* | 1.781189671 | 8.458285588 | 6.787741998 | III |
| *GmWRKY15* | 1.466540841 | 3.442338885 | 2.894047724 | I |
| *GmWRKY20* | 85.38603092 | 108.1304519 | 133.5087838 | III |
| *GmWRKY21* | 1.517028372 | 9.911962711 | 5.379598687 | I |
| *GmWRKY27* | 1.207748015 | 2.182487626 | 1.749404694 | IIb |
| *GmWRKY28* | 0.801153706 | 3.836362986 | 4.918738433 | III |
| *GmWRKY29* | 1.335854908 | 30.42100023 | 28.29030592 | IIc |
| *GmWRKY34* | 0.701201552 | 3.213128091 | 3.016166805 | IIb |
| *GmWRKY36* | 3.053106471 | 72.35103844 | 83.59687165 | IIc |
| *GmWRKY38* | 1.158935286 | 10.00251598 | 19.59956569 | III |
| *GmWRKY42* | 0.575023861 | 2.584892639 | 4.681361819 | IIb |
| *GmWRKY44* | 3.270389958 | 9.310001462 | 3.775598706 | IIc |
| *GmWRKY47* | 5.269435942 | 63.71533621 | 76.24184566 | IIc |
| *GmWRKY50* | 1.396542701 | 3.504654571 | 2.734527234 | IIe |
| *GmWRKY51* | 0.842581076 | 2.476139069 | 1.642329067 | III |
| *GmWRKY54* | 2.864964334 | 6.582618286 | 5.395426762 | IIa |
| *GmWRKY56* | 3.758454341 | 22.34396703 | 12.22123534 | III |
| *GmWRKY57* | 1.692718854 | 9.671407899 | 16.57029641 | III |
| *GmWRKY59* | 2.310438947 | 13.9636627 | 13.54854786 | IIc |
| *GmWRKY62* | 2.047645254 | 5.173645626 | 4.71293979 | IIb |
| *GmWRKY66* | 3.735186969 | 3.586816903 | 3.50304059 | IIb |
| *GmWRKY68* | 3.685163997 | 4.109740363 | 4.107683483 | IIa |
| *GmWRKY71* | 0.278599384 | 0.725076919 | 0.364668632 | IId |
| *GmWRKY74* | 2.240056117 | 2.655006173 | 3.96792373 | IIc |
| *GmWRKY75* | 0.886099786 | 4.453494502 | 4.924919878 | IIb |
| *GmWRKY76* | 7.304409461 | 128.9604763 | 50.06077873 | IIc |
| *GmWRKY77* | 2.109337886 | 3.40954519 | 2.29394749 | IIe |
| *GmWRKY78* | 1.413386149 | 4.511913905 | 2.53848146 | III |
| *GmWRKY81* | 2.125033244 | 8.804381892 | 4.750921598 | IIc |
| *GmWRKY82* | 0.783081294 | 1.467373862 | 2.001165333 | IIe |
| *GmWRKY83* | 1.652757783 | 8.058427258 | 10.49074546 | IIc |
| *GmWRKY85* | 3.809559248 | 4.72677016 | 3.53432116 | IIa |
| *GmWRKY89* | 1.752624535 | 3.472137787 | 3.478597493 | IIb |
| *GmWRKY93* | 0.504591387 | 1.199142415 | 2.360953117 | IIb |
| *GmWRKY100* | 1.834940201 | 2.218606057 | 2.320587943 | IIe |
| *GmWRKY101* | 2.795147537 | 9.246096746 | 6.589372296 | III |
| *GmWRKY102* | 0.764848248 | 2.991273763 | 1.776760995 | I |
| *GmWRKY107* | 2.240014948 | 2.891959878 | 1.306752065 | IIb |
| *GmWRKY111* | 0.878691821 | 6.862898718 | 7.197601916 | IIe |
| *GmWRKY114* | 1.826235692 | 6.7285942 | 8.05669672 | I |
| *GmWRKY115* | 2.206454344 | 5.093025385 | 4.979649366 | IIb |
| *GmWRKY119* | 2.136733234 | 13.50891361 | 10.54280454 | IIb |
| *GmWRKY120* | 3.7235332 | 9.39165857 | 9.631947821 | III |
| *GmWRKY125* | 2.775397856 | 4.675735103 | 4.398607773 | IIb |
| *GmWRKY126* | 8.572791543 | 20.05768303 | 20.62495604 | IIa |
| *GmWRKY134* | 17.84348555 | 225.5964503 | 130.6577921 | IIa |
| *GmWRKY140* | 1.242010208 | 2.528220465 | 2.292627475 | I |
| *GmWRKY141* | 1.494340162 | 2.141309989 | 1.892525834 | IIa |
| *GmWRKY142* | 1.076028949 | 3.470942369 | 3.311851369 | IIb |
| *GmWRKY146* | 1.085198744 | 1.675484258 | 2.681143673 | IIb |
| *GmWRKY147* | 1.203455978 | 3.644884873 | 4.063876118 | III |
| *GmWRKY150* | 2.632725525 | 10.07916346 | 10.19974775 | IIc |
| *GmWRKY153* | 5.000691471 | 10.10252999 | 13.67812381 | III |
| *GmWRKY154* | 1.518244438 | 2.247271129 | 2.493027315 | IIb |
| *GmWRKY155* | 2.278955076 | 2.074481661 | 2.322105656 | IIc |
| *GmWRKY156* | 3.099405781 | 5.02828224 | 5.1168695 | IIb |
| *GmWRKY159* | 0.73139766 | 3.349807644 | 2.988354768 | IIb |
| *GmWRKY163* | 0.726659088 | 13.68234023 | 7.863163256 | IIa |
| *GmWRKY164* | 6.348000318 | 25.19037564 | 31.77442402 | IIc |
| *GmWRKY166* | 3.760858374 | 3.760858374 | 5.297538456 | I |
| *GmWRKY171* | 1.30128053 | 3.887862426 | 2.676140262 | I |
| *GmWRKY172* | 2.463881102 | 12.91426829 | 11.10505131 | III |
| *GmWRKY179* | 1.219976657 | 2.988875476 | 2.953410972 | IIb |
| *GmWRKY180* | 3.444394667 | 23.49171474 | 25.53660111 | IIc |
| *GmWRKY183* | 2.100483373 | 2.243685719 | 2.798939341 | IIc |
| *GmWRKY185* | 1.40857882 | 8.358419806 | 8.040258441 | III |
