# Supplementary material for: Genome-wide identification of soybean WRKY transcription factors in response to salt stress
Source: Springerplus. 2016 Jun 29;5(1):920. doi: 10.1186/s40064-016-2647-x (PMC4927560; doi:10.1186/s40064-016-2647-x)
Supplement: Supplementary file 7 — 10.1186/s40064-016-2042-7 Normalized transcript levels of 49 GmWRKY genes in aerial part under salinity stress conditions. [file 40064_2016_2647_MOESM7_ESM.doc]

**Table S5.** Normalized transcript levels of 49 GmWRKY genes in aerial part under salinity stress conditions.

| Gene name | Transcript levels | group |
| --- | --- | --- |
| *GmWRKY2* | 0.44109216 | I |
| *GmWRKY5* | 0.46389533 | IId |
| *GmWRKY7* | 0.149192531 | III |
| *GmWRKY11* | 0.492255101 | I |
| *GmWRKY15* | 0.302922544 | I |
| *GmWRKY26* | 0.310099534 | IIc |
| *GmWRKY27* | 0.403515287 | IIb |
| *GmWRKY28* | 0.155026677 | III |
| *GmWRKY38* | 0.43879164 | III |
| *GmWRKY41* | 0.439070699 | IId |
| *GmWRKY45* | 0.45249378 | IIe |
| *GmWRKY47* | 0.146343685 | IIc |
| *GmWRKY51* | 0.295061674 | III |
| *GmWRKY54* | 0.183945216 | IIa |
| *GmWRKY55* | 0.41677729 | IId |
| *GmWRKY57* | 0.351486285 | III |
| *GmWRKY59* | 0.167550004 | IIc |
| *GmWRKY61* | 0.015070644 | IIc |
| *GmWRKY66* | 0.191501505 | IIb |
| *GmWRKY68* | 0.280343262 | IIa |
| *GmWRKY69* | 0.344009983 | III |
| *GmWRKY76* | 0.017282066 | IIc |
| *GmWRKY78* | 0.171423898 | III |
| *GmWRKY82* | 0.471856037 | IIe |
| *GmWRKY83* | 0.119140735 | IIc |
| *GmWRKY89* | 0.284891041 | IIb |
| *GmWRKY101* | 0.19540272 | III |
| *GmWRKY107* | 0.102036639 | IIb |
| *GmWRKY114* | 0.374029468 | I |
| *GmWRKY115* | 0.461314255 | IIb |
| *GmWRKY119* | 0.416816099 | IIb |
| *GmWRKY124* | 0.441747266 | IIe |
| *GmWRKY126* | 0.255065401 | IIa |
| *GmWRKY135* | 0.254846379 | IId |
| *GmWRKY140* | 0.283136653 | I |
| *GmWRKY141* | 0.155336525 | IIa |
| *GmWRKY142* | 0.139291699 | IIb |
| *GmWRKY146* | 0.080976718 | IIb |
| *GmWRKY147* | 0.196107485 | III |
| *GmWRKY155* | 5.076791809 | IIc |
| *GmWRKY157* | 0.470641555 | IId |
| *GmWRKY160* | 0.466020719 | IId |
| *GmWRKY161* | 0.391265795 | IId |
| *GmWRKY166* | 0.145561451 | I |
| *GmWRKY172* | 0.318397515 | III |
| *GmWRKY180* | 0.200022665 | IIc |
| *GmWRKY183* | 1.545059718 | IIc |
| *GmWRKY185* | 0.15944626 | III |
| *GmWRKY186* | 0.433316365 | I |
